# The use of neuralgia medications to treat sensory neuropathic cough: our experience in a retrospective cohort of thirty-two patients

**DOI:** 10.7717/peerj.816

**Published:** 2015-03-03

**Authors:** Zachary J. Bastian, Robert W. Bastian

**Affiliations:** 1 Department of Otolaryngology-Head and Neck Surgery, University of North Carolina, Chapel Hill, NC, USA; 2 Bastian Voice Institute, Downers Grove, IL, USA; 3 Department of Otolaryngology, Head and Neck Surgery, Loyola University Medical Center, Maywood, IL, USA

**Keywords:** Cough, Amitriptyline, Gabapentin, Treatment, Sensory neuropathy, Neuralgia, Desipramine

## Abstract

**Objective.** This study sought to: (1) quantify response rate and efficacy of amitriptyline, desipramine, and gabapentin in treating sensory neuropathic cough; and (2) describe an efficient treatment protocol.

**Study Design.** This study is a retrospective case series.

**Methods.** Persons diagnosed with sensory neuropathic cough during a one-year period were potential study candidates. To bolster the diagnosis credibility, only persons who had been treated elsewhere for gastroesophageal reflux disease, asthma, and allergy with no reduction of cough were included. Upon diagnosis of sensory neuropathic cough, each person was treated with either amitriptyline, desipramine, or gabapentin, titrating the dose upward to desired benefit or the dose limit. If the benefit was insufficient, another of the medications was used next, using a similar dose escalation strategy. Data points included patient demographics, initial and final medication, final dose, and degree of improvement.

**Results.** 32 patients met the diagnostic and inclusion criteria and had a complete data set. 94% (30 of 32) of the patients responded to at least one of the medications. The 32 patients undertook a total of 45 single-medication trials. Patients reported symptom relief during 78% (14 of 18) of amitriptyline trials, 73% (11 of 15) of desipramine trials, and 83% (10 of 12) of gabapentin trials. At final dosage, symptom reduction averaged 77% on amitriptyline, 73% on desipramine, and 69% on gabapentin.

**Conclusion.** Amitriptyline, desipramine, and gabapentin appear to vary in their effectiveness for individual cases of sensory neuropathic cough; across a whole cohort, symptom relief was similar in frequency and degree on any of the three medications. More evidence is needed to demonstrate more convincingly the effectiveness of these medications, but this data set suggests that each of these three medications deserves consideration in the codified treatment protocol presented here.

## Introduction

Coughing is responsible for 29.5 million visits to U.S. doctors every year (Irwin, 2006), commanding significant healthcare resources. Chronic cough can be due to diseases such as asthma, COPD, eosinophilic bronchitis, bronchiectasis, post-nasal drip, and gastroesophageal reflux disease. It also is a defining feature of sensory neuropathic cough (SNC).

SNC has been previously described, although called different names. Lee & Woo (2005) wrote about “sensory neuropathy presenting as chronic cough,” and they linked this type of chronic cough to an underlying sensory disturbance by demonstrating co-incident motor neuropathy using videostroboscopy and electromyography. Bastian, Vaidya & Delsupehe (2006) described SNC in detail as a clinical syndrome and proposed a set of diagnostic criteria. Vertigan & Gibson (2010) studied sensory disturbances and triggers (a feature of the SNC diagnosis) in a group of patients with refractory chronic cough. A review article by Gibson & Ryan (2011) includes sensory neuropathy in the diagnostic framework for chronic cough.

SNC sometimes develops following an upper respiratory infection, a herpes zoster outbreak in the pharynx, or thyroid or cervical spine surgery. Such cases provide insight into its mechanism. It behaves like vagal or glossopharyngeal neuralgia, but instead of pain, the nerve damage or dysfunction causes an intermittent, abrupt-onset sensation that initiates the coughing. In general, the sensation itself is virtually the same each time, though its perceived urgency may vary. Also common are patient-specific triggers such as talking, a loud laugh, attempting to sing a high note, taking a deep breath, breathing in cold air, touching a specific spot on the skin of the neck, and change of body position. While some cases have the apparent etiologies described above, a large number of patients develop SNC without an identifiable antecedent explanation.

This article reviews thirty-two sequential SNC patients in order to: (1) Compare the response rates and efficacy of three neuralgia medications (amitriptyline, desipramine, gabapentin) for treating SNC, and (2) Describe an efficient treatment protocol for SNC.

## Materials & Methods

Ethics approval (protocol #5460) for this case series was obtained from the institutional review board of Advocate Healthcare. Consent was waived for this retrospective study. All new patients diagnosed with SNC during the 12-month period spanning March 1, 2011 to February 28, 2012 were screened for inclusion. The SNC diagnoses had all been based on previously published criteria (Bastian, Vaidya & Delsupehe, 2006), including: cough duration of at least eight weeks; recurrent sensory disturbances of instantaneous onset (e.g., a tickle) immediately before each cough episode; neuralgia-like triggers (e.g., talking, cold air or warm air); dozens to hundreds of daily cough episodes; and non-productive cough. In none of these cases was there an attempt to obtain direct evidence of damage to sensory nerves, whether via clinical electrophysiology, biopsy, autopsy, or other means. No systemic evaluation of tic disorders or chronic somatization was performed, but patients we diagnosed with coughing tics or psychogenic cough were excluded from this series.

These SNC patients were screened for inclusion in this study based on the following additional and more stringent criteria:

• No other laryngologic diagnosis;
• Cough duration of at least five months;
• Prior treatment elsewhere for gastroesophageal reflux disease, asthma, and allergy as potential explanations for the chronic cough, using (respectively) proton pump inhibitors, asthma inhalers, and antihistamines, all with zero reported benefit.

These criteria produced 32 includable patients.

Table 1 details the treatment protocol for SNC developed and used by the senior author for over 15 years. Table 2 summarizes the various sequences of medication trials conducted among the group of 32 patients.

**Table 1:** Treatment protocol for sensory neuropathic cough.

Step 1: First-line medication trial
• Medication: amitriptyline or desipramine; amitriptyline for patients 60 years old or younger and, due to potentially lesser side effects, desipramine for patients older than 60 (as a general guideline)
• Trial method: Take 10 mg daily, two hours before bed, for the first day or two. Then, until symptoms are decreased 80% or more, or until a maximum dose of 80 mg is reached, continue increasing the daily dose by 10 mg every one or two days. If breakthrough cough is clearly evident in afternoon and evening, add 10 or 20 mg at noon.
Step 2: Phone follow-up, either 14 days after starting the medication, or sooner, if the patient has forgotten the instructions or has a concern
• At every phone follow-up, the patient must supply: name of current medication, dose, and duration of use; percent reduction of symptoms globally; side effects, if any; questions, if any; and best contact information. Staff records this information for physician review and response.
• Physician tells staff to instruct the patient to continue increasing dose (if the patient notices any reduction in cough symptoms whatsoever), possibly with instructions for adjusting the logistics of use (for instance, adding a booster of dose of 10 or 20 mg if the patient has breakthrough coughing at a certain time of day), or else to taper off current medication and then to begin another medication (if the current medication’s dose limit has been reached, or if side effects have become unacceptable, without providing satisfactory benefit to the patient).
Step 3: Second-line medication trial (if needed)
• Medication: gabapentin (best to take with food, except at bedtime)
• Trial method:
• Days 1–3: 300 mg at bedtime
• Days 4–6: 300 mg at lunch and bedtime (600 mg total)
• Days 7–9: 300 mg at breakfast, mid-afternoon, bedtime (900 mg total)
• Days 10–12: 300 mg at breakfast, lunch, dinner, bedtime (1200 mg total)
• After Day 12: If insufficient response, patient may (as side effects permit) add another 300 mg to one of the daily 300-mg doses every three days, up to a maximum daily dose of 2,400 mg (four 600-mg doses)
• Repeat Step 2.
Step 4: Third-line, fourth-line, fifth-line, etc. medication trials (if needed)
• Citalopram, pregabalin, oxcarbazepine
• Capsaicin spray^a^
Step 5: Gradual discontinuation trial (optional)
• An option for patients who achieve 80% or greater reduction of symptoms for at least two months
• Some patients will need long-term treatment, and some patients remit and relapse, with relapses often following upper respiratory infections.

**Notes.**

a Capsaicin spray is sometimes used for those who fail all medication trials. It is a topical spray administered to the posterior oropharynx. It appears to work either by gradually depleting substance p and thereby lowering the threshold for cough triggers, or as a counter-irritant to abort attacks in progress.

**Table 2:** Courses of the treatment trials. *n* = 32 patients; final medication tried in bold.

Number of patients	First medication	Second medication	Third medication
12	**Amitriptyline**		
7	**Desipramine**		
6	Desipramine	**Gabapentin**	
3^a^	Amitriptyline	**Gabapentin**	
1	Amitriptyline	Desipramine	**Gabapentin**
1	Amitriptyline	**Desipramine**	
1	Amitriptyline	Gabapentin	**Amitriptyline** + **gabapentin**
1	**Gabapentin**		

**Notes.**

a As mentioned in the footnote to Table 3, one of these patients went on to use capsaicin spray, with benefit.

Percent reduction of symptoms reported by the patient was this paper’s measure for degree of efficacy. Although not validated, it was published previously (Bastian, Vaidya & Delsupehe, 2006) to discuss the response of SNC to amitriptyline, and has been used by the senior author over at least 15 years of treating this entity.

During the course of a treatment trial, patients (often with third-party input) were asked periodically to report the percentage by which their original coughing symptoms had been reduced, considering together the original frequency, duration, and severity of their coughing episodes. The final assessment point was a minimum of six months after the start of the first neuralgia medication trial.

## Results

The cohort’s median age was 63, with an age range of 23 to 80 years. Female patients outnumbered male patients 3:1. The median duration of cough was 60 months, with a range of five to 432 months. The most common abrupt sensory disturbance preceding cough was a tickle experienced at the level of the sternal notch. An antecedent respiratory tract infection, remembered as specifically tied temporally to the onset of the cough, was reported by 38% of patients, with coughing continuing after the infectious symptoms resolved. Nighttime episodes awakening from sleep were reported by 28% of patients, and 16% reported at least occasional laryngospasm in association with severe episodes. Many patients also noted a crescendo of symptoms for several months after each subsequent upper respiratory infection.

Table 3 details patients’ final reported degree of response after one or more medication trials. The “no response” category includes one patient who had no response after trying amitriptyline and desipramine but who never tried gabapentin.

**Table 3:** Response quartiles, for patients at final medication and dose. *n* = 32 patients.

	No response	Subtle 1–25% reduction of symptoms	Small 26–50% reduction of symptoms	Medium 51–75% reduction of symptoms	Large 76–100% reduction of symptoms	**Total number**
Number of patients	2^a^	2	7	5	16	**32**
Percentage	6	6	22	16	50	**100**

**Notes.**

a One of these two patients went on to achieve 80% reduction of cough symptoms using capsaicin spray; note the footnote to Table 1 regarding capsaicin spray. The other patient tried amitriptyline and desipramine without benefit and never went on to a gabapentin trial.

Table 4 details the cohort’s response to individual medications. Responders were those who had a percent reduction of symptoms greater than 0. The median dose data shows that most patients needed to titrate upwards from initial dosing for maximal benefit. The data show response rates for amitriptyline and desipramine (usually given first) of 78% and 73%, respectively, and of 83% for gabapentin. They also show degrees of efficacy (by percent reduction of symptoms) ranging from 77% for amitriptyline to 73% for desipramine to 69% for gabapentin.

**Table 4:** Medication response. *n* = 45 treatment trials, taken by the 32 patients (some patients tried more than one medication) Excluded from this table is a 46th trial in which one of the patients, after trying amitriptyline and gabapentin separately, took amitriptyline and gabapentin together and achieved a 25% reduction of symptoms.

Medication	Number of patients who tried medication	Number/% who responded	Avg. % reduction of symptoms at final dose	Median final dose for responders (mg)
Amitriptyline	18	14/78	77	40
Desipramine	15	11/73	73	25
Gabapentin	12	10/83	69	1,350

## Discussion

Sensory neuropathic cough is diagnosed clinically, using the criteria mentioned above, and as the literature shows, it is effectively treated with neuralgia medications. Lee & Woo (2005) found gabapentin to be effective in the treatment of neuropathic chronic cough. Jeyakumar, Brickman & Haben (2006) did a randomized control trial using codeine plus guaifenesin versus the neuralgia medication amitriptyline for patients with a non-productive, intractable cough, and found amitriptyline to be superior. Halum, Sycamore & McRae (2009) reported a retrospective series in which pregabalin was effective for patients with laryngeal sensory neuropathy. A randomized, double blind, placebo-controlled trial done by Ryan, Birring & Gibson (2012) demonstrated gabapentin’s effectiveness for chronic refractory cough. Another paper described a cohort of patients with chronic idiopathic cough treated to good effect with gabapentin (Van de Kerkhove et al., 2012). Norris & Schweinfurth (2010) treated chronic cough patients with amitriptyline, using gabapentin second line, and found this regimen worked well.

Most patients in our cohort and in prior reports took amitriptyline at doses of only 10–40 mg daily (Jeyakumar, Brickman & Haben, 2006; Norris & Schweinfurth, 2010). Low-dose tricyclics are not uncommon in treating neuropathic pain (Moulin et al., 2014; Table 1), but most patients in controlled studies took much higher doses, e.g., ∼100–150 mg/d (Hearn et al., 2014; Derry et al., 2015). Similarly, duloxetine and venlafaxine are effective for neuropathic pain primarily at higher doses (Lunn, Hughes & Wiffen, 2014; Rowbotham et al., 2004). Since desipramine and amitriptyline have multimodal effects on the nervous system, their mechanism of action in SNC is not clear.

Left untreated, sensory neuropathic cough can profoundly affect a patient’s quality of life. While the dozens to hundreds of brief coughing episodes each day can annoy and frustrate, most patients also experience at least a few more severe and potentially humiliating episodes each day, lasting from 10 s to several minutes, and associated with rhinorrhea, oculorrhea, retching, vomiting, laryngospasm, near-syncope, and urinary incontinence. In the senior author’s practice, some patients have even reported cough-induced rib fracture, subconjunctival hemorrhage, and progressive pelvic floor weakening.

This disorder requires patience to treat and can be very labor-intensive. Many phone calls may be traded between patient and physician, with office staff serving as intermediaries, but typically a single visit and four or five episodes of subsequent phone management suffice. Patients who have become jaded or even contemptuous of clinician help after years of searching must be urged to persevere through one or more medication trials.

In this cohort, no medication emerged as clearly superior in the treatment of SNC, though we did not statistically verify that these medications’ efficacies were truly similar to one another, nor did we statistically analyze the sample sizes to determine what level of confidence could be placed in the effect of these medications. Interestingly, a paper comparing the efficacy of neuralgia medications for diabetic neuropathy among patients randomized and placebo-controlled to receive amitriptyline, duloxetine, or pregabalin also found that no one medication emerged as superior (Boyle et al., 2012).

This paper appears to be the first to present a clinical protocol for treating SNC. An effective treatment protocol must efficiently move the patient through a series of different medications, individualizing according to patient particulars, until a sufficient response is achieved. To accomplish this based upon a single patient visit, the physician carefully educates the patient and family up front through personal discussion, teaching videos, and written handouts, so that he or she can follow up effectively by phone. Regarding our gabapentin trial protocol, note that a QID interval works better in our experience than gabapentin’s usual TID interval, as many patients using a TID interval experience breakthrough coughing just before the next dose.

A prospective, randomized study with placebo controls would go further than our study to verify the relative efficacy of these three neuralgia medications. Still, as mentioned already, it should be noted that this cohort’s median cough duration was 60 months, during which numerous other medications (including treatments for gastroesophageal reflux disease, asthma, and allergy) had been tried for each patient reported here, without any perceived benefit. Also, the final assessment point was a minimum of six months after onset of treatment with medication for sensory neuropathy, and often much longer. Furthermore, others have employed randomization and a control group and found amitriptyline and gabapentin, respectively, to be effective for treating SNC (Jeyakumar, Brickman & Haben, 2006; Ryan, Birring & Gibson, 2012).

Efficacy data was based on patient report of overall percent reduction of symptoms, which was the only efficacy-related data available in this retrospective report. In the senior author’s experience, this form of patient response is easily understood by most patients and is clinically efficient and powerful, but it is a non-validated measure and less precise than some other cough measures, and thus is another limitation of this study.

## Conclusion

SNC is emerging as a distinct syndromic, diagnostic category for chronic cough. For this diagnosis, first-line agents amitriptyline and desipramine have response rates and degrees of efficacy that appear to be similar. Gabapentin also offers a comparable response rate, though with less convenient dosing. Thorough up-front education combined with phone follow-up is a good way to work with this labor-intensive group of patients, who often need more than one treatment trial with different neuralgia medications to achieve optimal benefit.

## Supplemental Information

10.7717/peerj.816/supp-1 Supplemental Information 1 Raw dataset of included cough patients
